# Spontaneous Osteogenic Differentiation of Human Mesenchymal Stem Cells by Tuna-Bone-Derived Hydroxyapatite Composites with Green Tea Polyphenol-Reduced Graphene Oxide

**DOI:** 10.3390/cells12111448

**Published:** 2023-05-23

**Authors:** Moon Sung Kang, Rowoon Park, Hyo Jung Jo, Yong Cheol Shin, Chang-Seok Kim, Suong-Hyu Hyon, Suck Won Hong, Junghwan Oh, Dong-Wook Han

**Affiliations:** 1 Department of Cogno-Mechatronics Engineering, College of Nanoscience and Nanotechnology, Pusan National University, Busan 46241, Republic of Korea; mskang7909@gmail.com (M.S.K.); rowoon.p153@gmail.com (R.P.); lisa0245@naver.com (H.J.J.); ckim@pusan.ac.kr (C.-S.K.); 2 Department of Inflammation and Immunity, Lerner Research Institute, Cleveland Clinic, Cleveland, OH 44195, USA; shiny2@ccf.org; 3 Engineering Research Center for Color-Modulated Extra-Sensory Perception Technology, Pusan National University, Busan 46241, Republic of Korea; 4 BMG Inc., Kyoto 601-8023, Japan; biogen@bmg-inc.com; 5 Industry 4.0 Convergence Bionics Engineering, Pukyong National University, Busan 48513, Republic of Korea; 6 Department of Biomedical Engineering, Pukyong National University, Busan 48513, Republic of Korea; 7 Ohlabs Corporation, Busan 48513, Republic of Korea; 8 BIO-IT Fusion Technology Research Institute, Pusan National University, Busan 46241, Republic of Korea

**Keywords:** bone tissue engineering, hydroxyapatite, reduced graphene oxide, human mesenchymal stem cell, osteogenic differentiation

## Abstract

In recent years, bone tissue engineering (BTE) has made significant progress in promoting the direct and functional connection between bone and graft, including osseointegration and osteoconduction, to facilitate the healing of damaged bone tissues. Herein, we introduce a new, environmentally friendly, and cost-effective method for synthesizing reduced graphene oxide (rGO) and hydroxyapatite (HAp). The method uses epigallocatechin-3-*O*-gallate (EGCG) as a reducing agent to synthesize rGO (E-rGO), and HAp powder is obtained from Atlantic bluefin tuna (*Thunnus thynnus*). The physicochemical analysis indicated that the E-rGO/HAp composites had exceptional properties for use as BTE scaffolds, as well as high purity. Moreover, we discovered that E-rGO/HAp composites facilitated not only the proliferation, but also early and late osteogenic differentiation of human mesenchymal stem cells (hMSCs). Our work suggests that E-rGO/HAp composites may play a significant role in promoting the spontaneous osteogenic differentiation of hMSCs, and we envision that E-rGO/HAp composites could serve as promising candidates for BTE scaffolds, stem-cell differentiation stimulators, and implantable device components because of their biocompatible and bioactive properties. Overall, we suggest a new approach for developing cost-effective and environmentally friendly E-rGO/HAp composite materials for BTE application.

## 1. Introduction

To date, many patients have experienced irreversible bone damage due to traumatic injuries, tumors, degenerative diseases, and bacterial or viral infections [1,2]. However, the supply of autograft and allograft is insufficient to meet clinical demands, and most artificial grafts do not provide proper cell–matrix interactions or mechanical synchronization with natural tissues, leading to weakening of the original tissues by shear stress and friction, or necrosis and inflammatory reactions [3]. Bone tissue engineering (BTE) scaffolds have emerged as a promising approach to rehabilitate bone losses by filling damaged cavities and facilitating native wound healing [4]. BTE scaffold development involves cytocompatible materials that are similar to those found in native bones, creating a microenvironment that promotes cellular adhesion, migration, proliferation, and osteogenic differentiation [5,6]. As a result, BTE studies use various biomaterials to support structural integrity and facilitate osseointegration, which is the direct and functional connection between bone and graft, as well as osteoconduction, which is the growth of new bone on the graft surface [7,8].

Hydroxyapatite (Ca_10_(PO_4_)_6_(OH)_2_ (Hap)) is a type of mineral apatite that contains hydroxyl, which can be synthesized using various methods, including precipitation [9], hydrothermal [10], mechano-chemical [11,12], polymer-assisted [13], and sol–gel techniques [14]. HAp has been highlighted for orthopedic applications such as bone defect fillers, bone graft substitutes, coating material of the graft surface, and extenders, particularly for its exceptional biocompatibility, osteoconductivity, and osteoinductivity [15,16]. Because the crystalline phase of natural bone is mostly composed of HAp (approximately 65 *w*/*w*%), its usage as a scaffold in BTE assures a strong affinity to the host-bone matrix and provides a native niche to promote the development of osteogenic phenotypes. Moreover, with a stoichiometric calcium-to-phosphorus ratio of 1:67 (*w*:*w*), HAp is the most stable and least soluble form of calcium phosphate composite in nature [17]. In particular, naturally derived HAp is known to exhibit more bioactivity compared with chemically synthesized HAp [18,19]. The superior bioactivity of naturally derived HAp comes from its nanometric crystal size and the presence of essential ions for bone morphogenesis, such as Mg^2+^, K^+^, Na^+^, and Sr^2+^ [18]. There are several kinds of HAp sources including eggshells, corals, fish bones, bovine, porcine bones, and even biowaste [19,20,21,22,23,24]. In particular, fish bone-derived HAp is apparently safer than that of mammalian bones as it does not have side effects such as bovine spongiform encephalopathy (BSE) and foot-and-mouth disease (FMD) [25]. Moreover, fish waste is a tremendous burden, and thus recycling it can lead to environmental advantages [26]. In this context, Atlantic bluefin tuna (*Thunnus thynnus*), which is a species of tuna in the family *Scombridae* found widely in the northern Pacific Ocean, was used as the HAp source [27]. Thermal calcination, which is a commonly used HAp extraction process, was used for the isolation of HAp from tuna bone [28].

However, the low mechanical strength of normal HAp ceramics restricts its use mainly to low load-bearing applications [29]. To overcome this issue, we incorporated reduced graphene oxide (rGO) nanosheets into HAp to increase the mechanical strength and osteogenic capability. rGO was prepared through the chemical or thermal reduction of GO to induce structural defects while maintaining oxidized groups [30]. The potential of rGO has been investigated as a prominent promoter for the osteogenic differentiation of human mesenchymal stem cells (hMSCs) [31]. The residual oxygen-containing functional moieties on the basal planes and edges of rGO actively absorb and interact with the surrounding biomolecules to modulate cell behavior [32]. In addition, our previous studies have demonstrated cell modulating effects of rGO, such as proliferation and osteogenesis, on the cell by utilizing graphene-based nanomaterials [33]. Furthermore, we utilized abundant polyphenols found in green tea, named epigallocatechin-3-*O*-gallate (EGCG), to synthesize rGO nanosheets, which is named E-rGO [34]. EGCG is known to be an effective and mild reducing agent for graphene materials; furthermore, it presents a π-conjugated structure that interacts with graphene sheets and helps with the disaggregation of graphene bundles to provide stable dispersions [35,36].

This study developed a new composite material named E-rGO/HAp composites by conjugating EGCG-reduced rGO with tuna-bone-derived HAp. The physicochemical properties of tuna-bone-derived HAp were found to be similar to those of pristine HAp. The morphology, chemical composition, and zeta potential of E-rGO/HAp were elucidated. Subsequently, cellular behaviors such as proliferation, ALP activity, and the formation of mineralization nodules were evaluated on E-rGO/HAp substrates.

## 2. Materials and Methods

### 2.1. Preparation of E-rGO/HAp Composites

#### 2.1.1. Preparation of E-rGO and Tuna-Bone-Derived HAp

**Figure 1:**
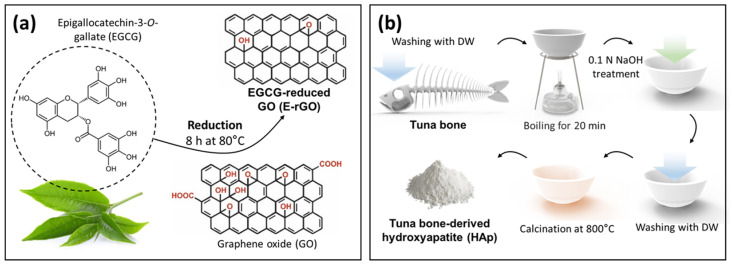
Schematic diagram of the preparation of E-rGO and tuna-bone-derived HAp. (**a**) Preparation process of E-rGO reduced by EGCG. (**b**) Procurement process of HAp from Atlantic bluefin tuna (*Thunnus thynnus*) bone.

The technique for converting GO into rGO involved heating it in a solution containing EGCG (BMG Inc., Kyoto, Japan). The procedure began by diluting the commercial GO solution (Sigma-Aldrich, St. Louis, MO, USA) to a concentration of 1 mg mL^−1^ in sterilized deionized (DI) water. Next, EGCG powder was added to the solution to achieve a final concentration of 10 mg mL^−1^. As shown in Figure 1a, the resulting mixture was tightly sealed and heated at 80 °C for 8 h. After heating, the mixture was sonicated for 1 h and gradually cooled down in water over a period of 12 h. Atlantic bluefin tuna (*Thunnus thynnus*) bone (Dongwon, Seoul, Republic of Korea) was cleansed thoroughly by washing with DI water to remove organic components. The bones were then boiled for 20 min and treated with 0.1 N NaOH, followed by rinsing with DI water. To purify HAp, the bones were calcined at 800 °C (Figure 1b). For the control, commercially available water-soluble HAp powder (Dentis Co., Ltd., Daegu, Republic of Korea) was used.

#### 2.1.2. Synthesis of E-rGO/HAp Composites

To prepare E-rGO/HAp composites, 1 mg mL^−1^ E-rGO as-prepared solution was sonicated for 2 h and then mixed with a 1 mg mL^−1^ tuna-bone-derived HAp powders suspended in DI water with a weight ratio of 1:1. Colloidal dispersions of E-rGO and HAp were vigorously mixed using a vortex for 10 min and slowly air-dried at 25 °C overnight, which resulted in the E-rGO/HAp composites.

### 2.2. Physicochemical Characterizations

#### 2.2.1. Physicochemical Characterizations of Tuna-Bone-Derived HAp

The composition of the tuna-bone-derived HAp was analyzed using Fourier-transform infrared (FT-IR) spectroscopy with a Spectrum GX instrument (PerkinElmer Inc., Waltham, MA, USA) attached. During the FT-IR analysis, the spectra were recorded in absorption mode and were scanned 16 times within the wavelength range of 500–4000 cm^−1^ with a resolution of 4.0 cm^−1^. To determine the crystalline structure of HAp, X-ray diffraction (XRD) patterns were obtained using an X-ray diffractometer (Empyrean series 2, PANalytical, Almelo, The Netherlands). HAp was scanned in the continuous scan mode using Cu-Kα radiation with a wavelength of λ = 0.154 nm, at a voltage of 40 kV and a current of 30 mA. Scanning was performed at a temperature of 25 °C in the 2θ range of 10–60°, at a scan rate of 2θ = 2° min^−1^. Thermogravimetry and differential scanning calorimetry (TG-DSC, STA 449 F1 Jupiter, NETZSCH-Feinmahltechnik GmbH, Selb, Germany) measurements were used to conduct a thermal analysis on both the pristine HAp and tuna-bone-derived HAp. The samples were heated to 800 °C at a rate of 5 °C min^−1^ in the presence of a helium gas flow, while the released gases were simultaneously analyzed by quadrupole mass spectrometry (403 Aëolos, NETZSCH-Feinmahltechnik).

#### 2.2.2. Physicochemical Characterizations of E-rGO/HAp Composites

The surface morphology of the prepared E-rGO/HAp composites was observed using FE-SEM (Carl Zeiss Supra 40VP, Oberkochen, Germany) at an accelerating voltage of 15 kV. The energy dispersive X-ray spectrometer (EDS) of the E-rGO/HAp composites was assessed using 10 images taken on random surfaces at 2000× magnification. Elemental mapping was performed for phosphorus, calcium, and carbon with a 10 mm^2^ window size, with a detectable range of 4Be-92U with a resolution of MnKα (131eV@100,000 count per second). The Raman spectra of the E-rGO/HAp composites were obtained using a Raman spectroscopy system (Ramboss 500i, Dongwoo Optron Co., Ltd., Gwangju, Republic of Korea) that included a charge-coupled device camera (iDusDV420A-OE, Andor Technology, Belfast, Ireland) and a precise motorized stage (SGSP 20–85, Sigma Koki Co., Ltd., Tokyo, Japan). An Ar-ion laser with a wavelength of 532 nm (LasNova 50, LASOS, Jena, Germany) was focused onto the sample by means of a water immersion objective lens (×60 magnification, numerical aperture of 1.2 UPlanSApo, Olympus Corporation, Tokyo, Japan) and the resulting signal was analyzed with a monochromator (Monora500i, Dongwoo Optron). The laser power was set at 5 mW at 532 nm and was attenuated using a 50% neutral density filter at the objective. The Raman spectra were collected over a range of 1200–2000 cm^−1^. The surface potentials of HAp, E-rGO, and E-rGO/HAp were measured using a zetasizer (Nano ZS, Malvern Instruments, Worcestershire, UK). While measuring the zeta potential, the pH value of each suspension (10 μg mL^−1^) was monitored and maintained at pH 7.0.

### 2.3. Cell Culture Conditions

Lonza (Walkersville, MD, USA) was the source of hMSCs used in all of the experiments, with cells between passages 3 and 5 being utilized. The hMSCs were regularly cultured at 37 °C under 5% CO_2_ in a humidified atmosphere using an MSC basal medium (BM, Lonza), supplemented with 10% MSC growth supplement (Lonza); 2% L-glutamine, 0.1% GA-1000; and 1% antibiotic-antimycotic solution (Sigma-Aldrich) that contained 10,000 units of penicillin, 10 mg of streptomycin, and 25 μg of amphotericin B per mL. When seeding the cells, the cell suspension was added with HAp, E-rGO, or E-rGO/HAp in BM at 10 μg mL^−1^ and then plated within 10 min to allow for growth in the monolayer.

### 2.4. Cell Proliferation Assay

To evaluate the impact of E-rGO/HAp composites on the proliferation of hMSCs, a cell counting kit-8 (CCK-8) assay was performed in accordance with the manufacturer’s guidelines (Dojindo Laboratories, Kumamoto, Japan). A concentration of 10 μg mL^−1^ of E-rGO/HAp composites were added to 48-well plates, and 1 × 10^4^ cells mL^−1^ of hMSCs were then seeded on the plates for predetermined durations. After 1, 3, and 5 days of incubation, each sample was washed twice with Dulbecco’s phosphate buffered saline (DPBS) and reacted with CCK-8 solution (diluted with DPBS at 1:9 *v*/*v*) for 2 h in a dark, humid environment at 37 °C under 5% CO_2_. The absorbance of each sample was measured at 450 nm using a microplate reader (Thermofisher scientific, Waltham, MA, USA) at each time point.

### 2.5. Alkaline Phosphatase (ALP) Activity Assay and Alizarin Red S (ARS) Staining

To investigate the hMSCs’ osteogenic differentiation, an ALP activity assay was conducted. The hMSCs were seeded at a concentration of 1 × 10^4^ cells mL^−1^ on 48-well plates, and 10 μg mL^−1^ of E-rGO/HAp was treated for 1, 7, 14, and 21 days. The conversion of *ρ*-nitrophenyl-phosphate to *ρ*-nitrophenol was measured to determine the ALP activity using an ALP assay kit (Abcam, Cambridge, UK) according to the manufacturer’s instructions. The absorbance at 405 nm was measured at each time point using a microplate reader. ALP activity was calculated by dividing the total amount of *ρ*-nitrophenol formation (μmol) by the reaction time (min) and sample volume (mL). To examine the formation of extracellular calcium deposits of the cells, hMSCs were seeded at a concentration of 1 × 10^4^ cells mL^−1^ on 48-well plates, and a 10 μg mL^−1^ of E-rGO/HAp was treated for 1, 7, 14, and 21 days. At each time point, the cells were washed twice with DPBS, fixed with 3.7% formaldehyde solution for 10 min, and then stained with 40 mM ARS dye (Sigma-Aldrich). The samples were then photographed with a digital camera (Olympus Corporation). To quantify the calcium deposits, the ARS dye was extracted from the stained cells by adding a 10% acetic acid solution and incubating the samples for 30 min with constant shaking at 80 rpm. A 10% ammonium hydroxide solution was then added to neutralize the aqueous solution of the ARS extracts, and the absorbance was measured at 405 nm using a microplate reader.

### 2.6. Statistical Analysis

All of the variables were tested in three independent cultures for each experiment, and then repeated twice (*n* = 6). The quantitative data are expressed as the mean ± standard deviation. Before the statistical analysis, data were analyzed for the equality of variances using Levene’s test. Multiple statistical comparisons were performed using the Bonferroni test after a preliminary one-way analysis of variance; the asterisks indicate statistical significance between groups (*** *p* < 0.001 and **** *p* < 0.0001), while not significant differences were not noted.

## 3. Results and Discussion

**Figure 2:**
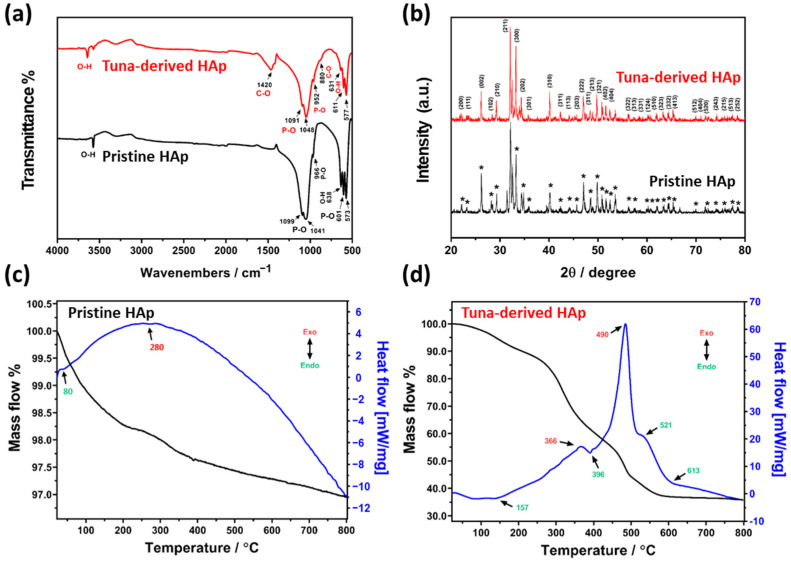
Physicochemical characterization of tuna-bone-derived HAp compared to pristine HAp. (**a**) FT-IR and (**b**) XRD spectra of tuna-bone-derived HAp and pristine HAp. Asterisks (*) indicate typical XRD peaks of pristine HAp. TG-DSC thermograms of (**c**) pristine HAp and (**d**) tuna-bone-derived HAp.

To validate the chemical compositions of pristine HAp and tuna-bone-derived HAp, the FT-IR spectra were analyzed (Figure 2a). On pristine HAp, the peak at 3568 cm^−1^ denotes ion stretching vibration of the O-H bond in the hydroxyl group [37]. The broad peaks at 1099, 1041, and 966 cm^−1^ represent the asymmetric stretching P-O bond in the phosphate ion [37]. The sharp peaks at 638, 601, and 573 cm^−1^ denote the asymmetric bending vibration of the P-O bond in phosphate ions [37]. On the other hand, characteristic peaks were observed in tuna-bone-derived HAp compared with pristine HAp. The peaks at 1420 and 880 cm^−1^ denote asymmetric stretching and the out-of-plane bending mode of the C-O bond in carbonate ions, respectively [38]. The XRD method was used to analyze the structure of a tuna-bone-derived HAp compared to a pristine HAp (Figure 2b). The sharp-shaped XRD peaks of the tuna-bone-derived HAp and pristine HAp indicate high crystallinity. The peak positions matched the JCPDS (896438) and corresponded with d-spacing values of 2.82 Å, 2.79 Å, and 2.72 Å, indicating a hexagonal system with a primitive lattice; these findings align with previous research [37,39]. Therefore, we confirmed the tuna-bone-derived HAp was successfully synthesized without impurities, with similar chemical characteristics as pristine HAp. Furthermore, we suggest that the extensive amount of oxygen-containing functional groups of tuna-bone-derived HAp could facilitate cellular behaviors by anchoring serum proteins and growth factors, as well as enhancing cell−matrix interactions [40].

Figure 2c,d display the TG-DSC thermograms of pristine HAp and tuna-bone-derived HAp, respectively. The mass flow curve of pristine HAp exhibits three main steps. The first step, occurring between 20 and 200 °C, shows a decrease in mass of around 1.7%, resulting from the evaporation of physically adsorbed water on the HAp surface. This step corresponds to the endothermic peak observed at approximately 60 °C in the heat flow [41,42]. The second step begins at around 250 °C and ends at 380 °C, causing a mass loss of roughly 0.7%, which can be attributed to the removal of chemically adsorbed water [41,42]. The DSC curve is dominated by an exothermic broad band, which corresponds to a structural rearrangement on the crystal lattice. At approximately 700 °C, continuous mass loss is observed due to the release of CO_3_^2−^, which occupies hydroxide or phosphate sites [43]. In contrast, the TG-DSC results of tuna-bone-derived HAp indicate a significant mass loss and chemical transformation. The first significant mass loss of 4.8% was recorded at 160 °C, which is associated with the massive dehydration of the sample and the elimination of the bone structure and collagen components [44]. The tuna-bone-derived HAp exhibits multi-stage losses up to 800 °C, which can be attributed to the removal of CO_3_, H_2_O, and NH_3_ content, suggesting the residual presence of matrix fiber organic compounds in the sample [45].

**Figure 3:**
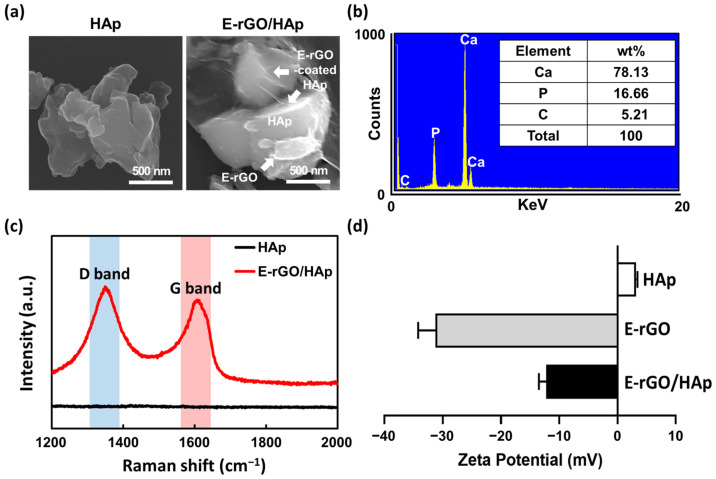
Physicochemical characterization of E-rGO/HAp compared to HAp. (**a**) SEM images of HAp and E-rGO/HAp, and (**b**) quantification of the EDS map on E-rGO/HAp. (**c**) Raman spectra of HAp and E-rGO/HAp. (**d**) Zeta potential measurement of HAp, E-rGO, and E-rGO/HAp.

Next, E-rGO/HAp was prepared as described in the Materials and Methods section using tuna-bone-derived HAp. The SEM images of E-rGO/HAp indicate that both HAp NPs and E-rGO HAp were approximately 500–800 nm in size and that single or few-layered E-rGO sheets were adsorbed on the surface of HAp NPs (Figure 3a). The EDS analysis shows that the E-rGO/HAp composites consisted of 78.13% calcium and 16.66% phosphate from the HAp components, along with 5.21% carbon from E-rGO (Figure 3b). The EDS spectrum did not reveal any other impurities within the detection limit of the instrument, which confirms the E-rGO/HAP composites’ purity, containing only Ca, P, and C elements. The semiquantitative analysis indicated a Ca/P molar ratio of approximately 4.69, which is significantly higher than the ratio observed in previous studies, typically around 1.66 [46]. This higher Ca ratio results in the CaO phase becoming the dominant phase in the HAp, which could enhance osteoblast adhesion [47].

The effectiveness of E-rGO conjugation with HAp was assessed using Raman spectroscopy of the HAp and E-rGO/HAp composites (Figure 3c). The Raman peaks at 1350 and 1610 cm^−1^ observed in the E-rGO/HAp composites were characteristic of carbon nanomaterials, specifically the D band (~1350 cm^−1^) and G band (~1600 cm^−1^) [48,49]. The G band indicates hybrid carbon from graphene, while the D band arises from structural defects from sp^2^ hybrid carbon [50]. The I_D_/I_G_ intensity ratio was found to be 1.11, suggesting that the carbon nanomaterial is rGO and its original chemical characteristics were retained [51]. The zeta potential analysis of the surface potentials shows that the E-rGO in DI water (pH 7.0) were charged at about −32.1 mV, whereas HAp microparticles were charged at about +4.3 mV (Figure 3d). It was shown that E-rGO was negatively charged over a very wide pH range (0.5–10.0) and their zeta potential fluctuated between −30 mV and −25 mV after 6 h or more of hydrazine reduction at pH 7.0 [52]. A zeta potential >30 mV (absolute value) is generally regarded as a critical value that represents sufficient mutual repulsion to guarantee the stability of a dispersion [53]. The surface charge of E-rGO/HAp composites was measured to be around −13.6 mV. These results indicate that E-rGO/HAp composites were formed via electrostatic interactions between HAp particles and E-rGO.

**Figure 4:**
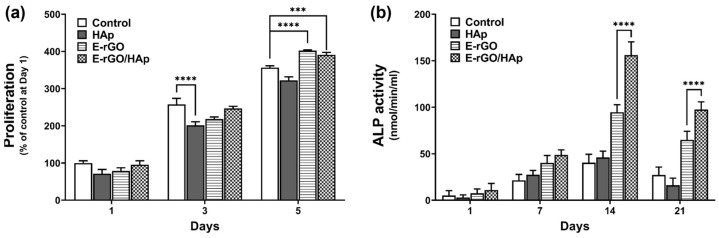
Effects of E-rGO/HAp composites on the proliferation and ALP activity of hMSCs. (**a**) Proliferation of hMSCs cultured with HAp, E-rGO, and E-rGO/HAp. (**b**) ALP activity of hMSCs cultured with HAp, E-rGO, and E-rGO/HAp. The asterisks represent statistical differences between groups (*** *p* < 0.001 and **** *p* < 0.0001, *n* = 6).

Maintaining the active proliferation of stem cells is crucial for bone-tissue engineering scaffolds. To assess the proliferation of hMSCs, cells were cultured with HAp, E-rGO, and E-rGO/HAp for 5 days (Figure 4a). After 3 days, the cell proliferation in the HAp group was significantly reduced (77%) compared with the control group, while there were no significant differences in the E-rGO and E-rGO/HAp groups. However, after 5 days, the proliferation of the E-rGO and E-rGO/HAp groups was significantly increased (112% and 110%, respectively) compared with the control group. Therefore, it is demonstrated that HAp can decrease the early cell proliferation of hMSCs, and the addition of E-rGO can not only mitigate this effect, but also increase cell proliferation over longer culture periods. Osteoblasts synthesize and secrete type-I collagen, ALP, and other enzymes that regulate mineral deposition, leading to the formation of hydroxyapatite crystals and the mineralization of bone tissue [54]. To compare the ability of HAp, E-rGO, and E-rGO/HAp to promote osteogenic differentiation, the ALP activity of hMSCs was assessed in Figure 4b. All of the groups showed the highest ALP activity at 14 days, which tended to decrease at 21 days. This is consistent with the observation that ALP is an early osteogenesis marker and is predominantly expressed during hMSCs differentiation into osteogenic lineages [55]. In particular, the hMSCs in the E-rGO and E-rGO/HAp groups showed increased ALP activity at 14 and 21 days compared with the control and HAp groups. Furthermore, the ALP activity of the E-rGO/HAp group was significantly increased compared with the HAp group (164% at day 14 and 152% at day 21), indicating the synergistic osteogenic effects of E-rGO and HAp.

**Figure 5:**
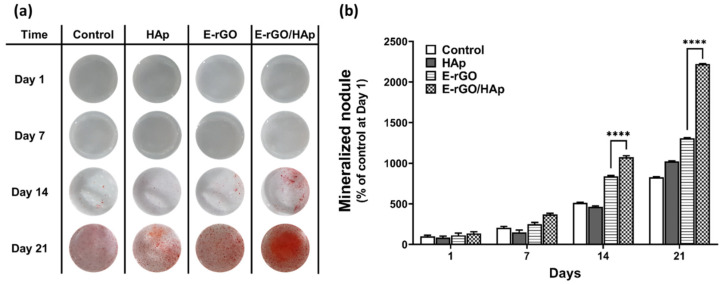
Effects of E-rGO/HAp composites on calcium deposition and matrix mineralization in hMSCs. (**a**) Digital images of ARS staining and (**b**) quantification of mineralized nodule formation on hMSCs cultured with HAp, E-rGO, and E-rGO/HAp. The asterisks represent statistical differences between groups (**** *p* < 0.0001, *n* = 6).

To further explore the osteogenic-differentiation-inducing effect of E-rGO/HAp composites, calcium phosphate deposition, which is considered as a later marker for bone regeneration, was observed through ARS staining. The image of ARS staining (Figure 5a) and its quantification (Figure 5b) showed that E-rGO and E-rGO/HAp groups showed increased mineralization nodule formation compared with the control. Furthermore, the formation of mineralized nodules in the E-rGO/HAp group was significantly (*p* < 0.0001) increased compared with the E-rGO group (129% at day 14 and 171% at day 21), suggesting the E-rGO/HAp composites can remarkably induce an osteoid matrix deposition, even without any osteogenic-inducing agents.

Numerous studies have demonstrated the osteogenic effects of HAp derived from unique aspects of the material [56,57]. Some studies attribute HAp’s osteogenic effects to geometric factors, such as the micropores in HAp ceramics that connect macropores, permitting interstitial fluid flow through the matrix [58]. At the initial stage of cell adhesion, the surface of HAp can affect gene expression, and signal transduction pathways depend on the attachment of stem cells to HAp surfaces [59]. This attachment leads to the sequential expression of integrins, FAK, and ERK genes required for cell attachment, followed by the expression of proliferative genes, c-jun and c-fos genes, culminating in the expression of the ALP gene during the differentiation stage [60]. The exposure of HAp in hMSC is also known to induce CD10 and CD90 expression, which are commonly reported as up-regulated markers in osteoblasts [61,62]. Furthermore, HAp induces the expression of CD105, a modulator of cellular response upon stimulation with TGF-β, indicating hMSCs are differentiated into osteogenic lineages [63,64]. The introduction of HAp leads to an increase in bone morphogenetic protein 2 (BMP-2), which in turn boosts the expression of Runt-related transcription factor 2 (RUNX2), a critical transcription factor responsible for triggering osteogenic differentiation. This upregulation also results in the expression of various major bone matrix genes, including osteocalcin (OCN), osteopontin (OPN), bone sialoprotein (BSP), and ALP [61].

Despite its potential as a cell scaffold, HAp has some drawbacks, such as low electrical conductivity and brittleness. To address these limitations, rGO can be added to HAp scaffolds to enhance their electrical conductivity, which in turn allows for the adsorption of serum proteins and growth factors to the scaffold’s numerous surface functional groups, such as hydroxyl, epoxy, carbonyl, and carboxyl groups [65]. This effect increases the local concentration of proteins and osteogenic inducers on the scaffold surface, which are then available for stem cells. The microporous structures and structural defects of the resulting E-rGO/HAp composites are suggested to benefit the growth and osteogenic differentiation of stem cells by providing an extracellular matrix (ECM) biomimetic microenvironment [66]. Some previous studies have proposed several underlying mechanisms for the ability of rGO to induce the osteogenic differentiation of hMSCs. It has been reported that the incorporation of rGO into scaffolds increases the expression levels of key osteogenic markers such as BSP, OCN, OPN, and RUNX2 [67]. These graphene-based composites activate diverse osteogenic pathways, including the Wnt-related pathway, Hif-1α pathway, and BMP-2 upregulation via the Erk1/2 signaling pathway [68,69]. Furthermore, the addition of rGO to HAp scaffolds improves their mechanical strength, which promises greater opportunity for orthopedic applications [70]. Therefore, we suggest that the addition of E-rGO to HAp scaffolds enhances their osteogenic differentiation-inducing effect, supporting the early and late osteogenic differentiation of hMSCs. This approach provides a promising strategy for addressing the low electrical conductivity and brittleness of HAp, while also offering improved mechanical strength and a biomimetic microenvironment for the growth and osteogenic differentiation of hMSCs.

## 4. Conclusions

To summarize, we introduced environmentally friendly and cost-effective methods for synthesizing E-rGO and tuna-bone-derived HAp. E-rGO was reduced using the green tea extract EGCG, while HAp powder was obtained from Atlantic bluefin tuna (*Thunnus thynnus*). The physicochemical analysis indicated that the E-rGO/HAp composites had exceptional chemical properties for use as a tissue engineering scaffold, as well as high purity. Moreover, we discovered that culturing hMSCs with E-rGO/HAp composites induced spontaneous osteogenic differentiation. However, more detailed mechanisms involving intracellular signaling pathways remains obscure and require further study at a molecular level. Nonetheless, our work suggests that E-rGO/HAp composites may play a significant role in promoting the spontaneous osteogenic differentiation of hMSCs, and we envision that these E-rGO-based composites could serve as promising candidates for tissue engineering scaffolds, stem cell differentiation stimulators, and implantable device components due to their biocompatible and bioactive properties.

## Data Availability

All data needed to evaluate the conclusions in the paper are present in the paper. Additional data related to this paper may be requested from the authors.
